# Prolapsus of the Uterus in the Bitch

**Published:** 1900-02

**Authors:** C. S. Gelbert

**Affiliations:** Scranton, Pa.


### PROLAPSUS OF THE UTERUS IN THE BITCH.

By C. S. Gelbert, V.M.D.,
SCRANTON, PA.

Prolapus of the vagina is more common than prolapsus of the uterus. In some instances it is accompanied by serious alterations of the vagina, especially hypertrophic alterations, and also, in rare cases, by polypus formations. These alterations are generally caused by difficult whelping. As a rule, there is more or less protrusion of the vagina through the vulva, appearing in the form of pear-shaped or flap-shaped, red, inflamed tissue, covered with mucus. In very rare instances the prolapsus is so great that the os of the uterus can be seen through the external opening. When the uterus is prolapsed the protruded portion is forced out of the vulva, and we see a pear-shaped body, intensely red, with salient borders. *One horn of the uterus is protruded only.*

I was called in to see a bitch on January 24th, which had given birth to six puppies five days before. On examining the animal, which was very much emaciated, I noticed a Y-shaped body (protrusion of both horns) protruding from the vulva, in length about five inches and three inches in circumference. It was much congested and quite firm to pressure; decomposition had set in, and a foul odor was given off. The mammæ were quite dry, and the animal was in a sad plight. Temperature taken per rectum was 102.6° F.; pulse fast and irregular; respirations 12 per minute. I decided that an operation was necessary, but had little hope of saving her life.

*Operation.* Disinfected the affected organ and its surroundings with a 5 per cent. creolin solution, ligated with a silk thread two and a half inches anterior to the os uteri, to prevent hemorrhage and infection. I then cut through the vagina one inch in front of the os uteri. The scalpel went through a mass of fat surrounding the body of the uterus and then through the ovarian ducts. There was a slight hemorrhage. After thorough antisepsis the stump of the vagina was returned to its natural place. There was some pain when the ligature was applied before the operation, but during the operation the animal did not show the least sign of pain. Gave camphor to stimulate the heart and calomel and sodi bicarbonatis to keep the bowels open. The day after the operation her temperature was 104.2° F., and on the second day she died of septic peritonitis.

